# Thermal Degradation Kinetics of Anthocyanins Extracted from Purple Maize Flour Extract and the Effect of Heating on Selected Biological Functionality

**DOI:** 10.3390/foods9111593

**Published:** 2020-11-03

**Authors:** Mioara Slavu (Ursu), Iuliana Aprodu, Ștefania Adelina Milea, Elena Enachi, Gabriela Râpeanu, Gabriela Elena Bahrim, Nicoleta Stănciuc

**Affiliations:** Faculty of Food Science and Engineering, Dunarea de Jos University of Galati, 111 Domnească Street, 800201 Galați, Romania; ursumioara@ymail.com (M.S.); Iuliana.Aprodu@ugal.ro (I.A.); Adelina.Milea@ugal.ro (Ș.A.M.); Elena.Ionita@ugal.ro (E.E.); Gabriela.Rapeanu@ugal.ro (G.R.); Gabriela.Bahrim@ugal.ro (G.E.B.)

**Keywords:** purple maize, anthocyanins, antioxidant activity, thermal treatment, in vitro release, molecular modeling

## Abstract

The thermal degradation of the anthocyanins and antioxidant activity in purple maize extracts was determined between 80 and 180 °C. The anthocyanins were found to be thermostable in the temperature range of 80 to 120 °C, whereas at higher temperatures the thermal degradation of both anthocyanins and antioxidant activity followed a first-order kinetic model. The *z*-values started from 61.72 ± 2.28 °C for anthocyanins and 75.75 ± 2.87 °C for antioxidant activity. The conformational space of pairs of model anthocyanin molecules at 25 and 180 °C was explored through a molecular dynamics test, and results indicated the occurrence of intermolecular self-association reactions and intramolecular co-pigmentation events, which might help explaining the findings of the degradation kinetics. The relationship between thermal degradation of anthocyanins and antioxidant activity and the in vitro release was further studied. The unheated extracts showed a high stability under gastric environment, whereas after heating at 180 °C, the digestion ended quickly after 60 min. After simulated intestinal digestion, the anthocyanins were slowly decreased to a maximum of 12% for the unheated extracts, whereas an 83% decrease was found after preliminary heating at 180 °C. The thermal degradation of anthocyanins was positively correlated with the in vitro decrease of antioxidant activity.

## 1. Introduction

It has been suggested that food color is the one of the most important visual indicators of taste and flavor, as both are considered vital sensory properties of food [1]. In recent years, considerable effort has been made to identify alternatives for synthetic dyes in food and beverages with natural colorants due to the public concern regarding their potential adverse effects including potential adverse behavioral and neurological effects and hyperactivity in children [2]. Although these studies have not established a concrete link between the intake of synthetic dyes and detrimental health effects, still a general move toward “more natural ingredients” is forcing the food processing industry to explore natural replacements for synthetic dyes [3].

Anthocyanins (ANCs) are well known and currently intensely studied as sourced from dark-colored fruits and vegetables such as black grapes, blueberries, and purple carrots [4], in addition to their health promoting functions, such as: antioxidant and anti-cancer [5], and anti-obesity and anti-inflammation effects [6]. However, ANCs can be involved in chemical and enzymatic reactions that may degrade them to colorless products or transform them into new structures [7]. Besides this, their health promoting effects are also hindered by their low stability under the physicochemical conditions they are exposed to after oral consumption by humans, affecting their bioaccessibility and further bioavailability [8]. Thus, when considering the use of ANCs as food pigments and health promoting ingredients, their thermal treatment, enzymes, co-pigmentation, and the pH instability should be considered. These types of processing and factors might induce changes that are likely to significantly change ANCs’ concentration and bioactivity, which further affects consumer acceptance of a product.

Thermal treatment is one of the most commonly used methods to preserve and extend the shelf life of foods in order to ensure food safety [9], involving heating in the temperatures range between 50 and 180 °C, depending on the characteristics of the product and the desired shelf life. Thus, it is expected that processing at different temperature–time combinations specific to unit operations affects the color, anthocyanin content, and the antioxidant capacity of the ANCs present in a food [10]. Additionally, associated with each thermal process, degradation of heat-sensitive vitamins, proteins, anthocyanins, conversion to colorless derivatives and subsequently to insoluble brown pigments, and changes in structure and other quality factors that are undesirable, should be considered. In general, the thermal processing must be drastic/strong enough to destroy the microorganisms and degrading enzymes, but at the same time, it should be mild enough to avoid chemical changes that might impair the food’s flavor and nutritional value [11]. Therefore, accurate knowledge of the kinetic parameters is essential to predict the quality changes that occur during thermal processing.

Colored corn is one of the richest sources of ANCs, with concentrations ranging from 51 mg cyanidin-3-*O*-glucoside equivalent (C3G)/kg in red corn to 1300 mg C3G/kg fresh weight (fw) in purple corn [12]. Li et al. [13] suggested that large-scale economical cultivation of corn and its long shelf life makes it a very attractive source for efficient ANC extraction. Colored corn contains a number of different anthocyanin species including cyanidin-, pelargonidin-, and peonidin-based monoglucosides, malonyl and dimalonyl glucosides, and flavonol anthocyanin condensed forms.

Therefore, the aim of this study was to advance the knowledge on anthocyanins from the purple maize extract obtained by combining solvent- and ultrasound-assisted extraction, based on the total monomeric and individual anthocyanin profiles. Further, since accurate knowledge of the kinetic parameters is essential to predict the quality changes that occur during thermal processing of different foods, our study reports the degradation kinetic parameters of ANCs and related antioxidant activity during heating at various temperatures ranging from 80 and 180 °C for different heating times (0–40 min). Furthermore, studies involved establishing a link between ANCs’ thermal degradation at different selected time–temperature combinations with the effect of in vitro digestion on ANCs and antioxidant activity.

## 2. Materials and Methods

### 2.1. Chemicals

Gallic acid, catechin, Trolox (6-hydroxy-2,5,7,8-tetramethylchromane-2-carboxylic acid), Folin–Ciocalteu’s reagent, pepsin from porcine gastric mucosa (≥400 units/mg protein), and pancreatin from porcine pancreas (1400 FIP-U/g protease, 24,000 FIP-U/g lipase, 30,000 FIP-U/g amylase) were purchased from Sigma Aldrich Steinheim (Darmstadt, Germany). The standards used for the HPLC analysis, namely cyanidin-3-glucoside, pelargonidin-3-glucoside, and peonidin-3-glucoside, were acquired from Sigma Aldrich Steinheim (Darmstadt, Germany). All other chemicals and reagents used in the experiments were of analytical grade.

### 2.2. Plant Material

The purple maize flour (*Zea mays* L.) was generously supplied by a local producer (Brăila County, Romania), in September 2019. The flour with a water content <12% was sealed in a brown bottle and kept at 4 °C until analysis. The flour was sifted through a 100 mesh sieve and used for further anthocyanin extraction.

### 2.3. ANC Extraction

An amount of 10 g of sieved flour was extracted with 90 mL of 70% ethanol and 10 mL of HCL 1N. The extraction was performed using a sonication water (MRC Scientific Instruments, Holon, Israel) bath for at 40 °C for 30 min, followed by centrifugation at 5000× *g* for 10 min at 4 °C. The supernatant was collected, and the extraction was repeated three times. After extraction, the collected supernatants were pooled together and concentrated under reduced pressure at 40 °C, using a vacuum rotary evaporator (AVC 2-18, Christ, UK). The obtained extract was dissolved in ultrapure water, characterized, and used for kinetic experiments.

### 2.4. Total Monomeric ANC Content

The total monomeric anthocyanin content (TAC) was determined according the pH-differential method, as explained by Giusti and Worsltad [14]. An aliquot of the extract was dissolved in 1 mL of distillated water. Aliquots of 0.2 mL were added to 0.8 mL of buffers with of pH 1.0 and 4.5, respectively. Absorbance was measured by a spectrophotometer (Biochrom Libra S22 UV/Vis, Cambridge, UK) at 520 and 700 nm, respectively. Absorbance was calculated as Abs = (A_520_ − A_700_)_pH 1.0_ − (A_520_ − A_700_)_pH 4.5_ with a molar extinction coefficient for cyanidin-3-glucoside of 26,900 L⋅mol^−1^⋅cm^−1^. TAC was calculated using the following equation and expressed as milligrams of cyanidin-3-*O*-glucoside equivalents per g dry weight (mg C3G/g DW) (Equation (1)).

(1) $$TAC\;\left(mg\frac{C3G}{gDW}\right)=\frac{Abs}{eL}M_{w}\times D\times \frac{V}{M}$$

where *Abs* is absorbance, *e* is cyanidin-3-*O*-glucoside molar absorbance (26,900 L⋅mol^−1^⋅cm^−1^), *L* is the cell path length (1 cm), *M_W_* is the molecular weight of anthocyanin (449.2 Da), *D* is the dilution factor, *V* is the final volume (mL), and *M* is the dry weight (mg) [15].

### 2.5. Chromatographic Analysis of ANCs

To obtain the chromatographic profile of the anthocyanins from purple maize flour extract, an HPLC analysis was conducted using a Thermo Finnigan HPLC system (Thermo Scientific, Waltham, MA, USA). In order to separate and identify the compounds, a Synergi 4u Fusion-RP 80A (150 × 4.6 mm, 4 μm) column was used, monitored at 520 nm wavelength, at an oven temperature of 27 °C. Firstly, the samples were filtered through a C18 Sep-Pak cartridge (Waters), in order to remove the unwanted compounds, and secondly through a 0.22 μm syringe filter (Bio Basic Canada Inc., Markham, ON, Canada). The two solvents that were used for the elution step were 10% formic acid (A) and 100% methanol (B). The volume of the injection was 10 μL, at a flow rate of 1.0 mL/min. The anthocyanins from the purple maize flour extract were identified based on the retention time of the standard compounds and the data reported in the literature.

### 2.6. DPPH Radical Scavenging Analysis

A volume of 0.1 mmol solution of methanolic DPPH solution was prepared. The initial absorbance of the DPPH in ethanol was measured at 515 nm and did not change throughout the period of assay. Aliquots (0.1 mL) of each sample were added to 2.9 mL of DPPH solution. Discolorations were measured at 515 nm after incubation for 60 min at 25 °C in the dark. Working solutions of Trolox (range from 0 to 10 mmol) were used for calibration. The antioxidant capacity was calculated from the linear calibration curve expressed as mmol Trolox/g DW [15].

### 2.7. Thermal Treatment

Aliquots of 2.0 mL purple maize flour anthocyanins extract were put into screw-cap test tubes already equilibrated in a digital block heater (Stuart) at temperatures ranging from 80 to 180 °C, with an increase of 10 °C. At regular time intervals (2, 5, 7, 10, 20, 30, and 40 min), samples were removed from the bath and rapidly cooled by plunging into an ice water bath. The analysis was conducted immediately.

### 2.8. Kinetic Parameters

Data for the change in ANC content and antioxidant activity over time were fitted to a first-order degradation kinetics model. The kinetic rate constant (*k*) of thermal degradation, decimal reduction time (*D*), *z*-values, and activation energy (*E_a_*) were defined as described by Peron et al. [16].

### 2.9. Static In Vitro Digestion

The simulated in vitro digestion model was prepared using a modified method described by Lee et al. [17], involving gastric and intestinal fractions. The purple maize flour anthocyanin extracts (equivalent to 10 mg C3G/g DW) were dissolved in simulated gastric juice (containing 100 mL HCl and 100 mg pepsin, pH 2.0) and digested sequentially at 37 °C using a water bath for 2 h. Aliquots of 2 mL were collected, centrifuged, filtered through a 0.22 μm filter, and analyzed for TAC and antioxidant activity at every 30 min. After 2 h, 5 mL of the digested sample was mixed with 10 mL of simulated intestinal juice (100 mL NaHCO_3_ and 200 mg pancreatin, pH 7.0) and digested for another 2 h. The samples were prepared in triplicate (*n* = 3).

### 2.10. Single-Molecule-Level Investigations on Anthocyanin Behavior at Thermal Treatment

Molecular modeling techniques were further employed to simulate the heat-induced behavior of main anthocyanins from purple maize, as indicated by the HPLC analysis. The Hyperchem 8.0 software (Hypercube Inc., Gainesville, FL, USA) was used to prepare and optimize the molecular models of cyanidin-3-*O*-glucoside and its acylated form cyanidin-3-*O*-(6″-malonylglucoside). The geometry optimization of these molecules was performed using in sequence the steepest descent and conjugate gradient algorithms. The optimized molecules, obtained when getting potential energy gradients lower than 0.001 kcal/Å·mol, were further used as models for investigating the thermal-dependent self-association and intramolecular copigmentation reactions. In order to study the effect of the thermal treatment on the anthocyanins’ behavior, six different starting models, consisting of anthocyanins of the same type, were prepared so as to favor all types of self-associations, in agreement with poses proposed by Castaneda-Ovando et al. [18]. The starting models were obtained by merging two anthocyanin molecules of the same type, so as to get the following three complexes: complex 1—having the oxygen of benzopyrylium of one anthocyanin molecule interfaced with the phenyl aromatic ring of the second anthocyanin molecule; complex 2—obtained by placing the oxygen atoms of the phenyl aromatic ring of one anthocyanin molecule nearby one of the hydroxyls of benzopyrylium of the second anthocyanin molecule; complex 3—obtained by interfacing the hydroxyls of benzopyrylium of one anthocyanin molecule with the sugar moiety of the second anthocyanin molecule [19]. The orientation and the distance of about 1.5 Å between functional groups of the two molecules of each complex were chosen so as to allow possible hydrogen bonding. Molecular dynamics steps were further employed to heat and equilibrate each of the six starting models at 25 and 180 °C for 100 ps. The equilibrated models were characterized in terms of atomic-level details important for predicting eventual heat-dependent self-association or co-pigmentation events.

### 2.11. Statistical Analysis

All analyses were conducted in at least three independent replicates, and the results are reported as mean ± standard deviation. Statistical comparisons were made by one-way analysis of variance (ANOVA). Differences were considered to be significant when the *p* values were <0.05. The parameters of kinetic models and Arrhenius equation were estimated by linear regression. Mathematical models were selected by comparing correlations coefficients.

## 3. Results

### 3.1. Characterization of the Extract

It is well known that the concentration of ANCs may vary among foods produced by a given plant species due to different external and internal factors, such as genetic and agronomic factors, intensity and type of light, temperature, processing, and storage, as explained by de Pascual-Teresa et al. [20]. In our study, the purple maize flour extract showed a total monomeric anthocyanin content (TAC) content of 520.42 ± 23.88 mg C3G/g DW. Our results are similar with those reported by Li et al. [13], who obtained a TAC from purple corn extract of 4933.1 ± 43.4 mg C3G/kg dry corn, whereas Saikaew et al. [21] showed a TAC of the untreated purple kernels of 101.76 ± 1.16 mg CGE/100 g DW, respectively.

**Figure 1:**
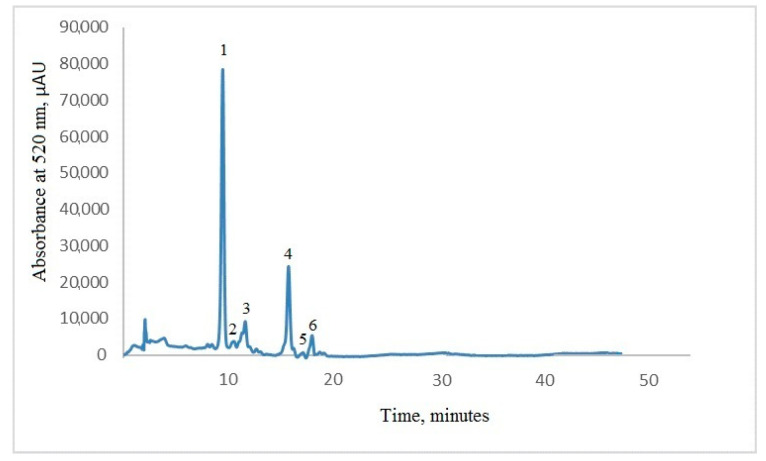
Chromatographic profile of the purple maize flour extract: Peak 1—cyanidin-3-*O*-glucoside; Peak 2—pelargonidin-3-*O*-glucoside; Peak 3—peonidin-3-*O*-glucoside; Peak 4—cyanidin-3-*O*-(6′′-malonylglucoside); Peak 5—pelargonidin-3-*O*-(6′′-malonylglucoside), and Peak 6—peonidin-3-*O*-(6′′-malonylglucoside).

The ANC composition of purple maize flour was determined by HPLC at 520 nm. The HPLC-DAD profile of the purple maize flour extract displayed the presence of six main compounds such as cyanidin-3-*O*-glucoside, pelargonidin-3-*O*-glucoside, peonidin-3-*O*-glucoside, cyanidin-3-*O*-(6″-malonylglucoside), pelargonidin-3-*O*-(6″-malonylglucoside), and peonidin-3-*O*-(6″-malonylglucoside) (Figure 1). The two major compounds were cyanidin-3-*O*-glucoside and its acylated form cyanidin-3-*O*-(6″-malonylglucoside), with a concentration that represented 47% of the total anthocyanin content for cyanidin-3-*O*-glucoside, while the concentration of cyanidin-3-*O*-(6″-malonylglucoside) accounted for 18% of the total anthocyanins content. Nonetheless, the major anthocyanin, cyanidin-3-*O*-glucoside, registered a concentration of 9.85 mg/g DW. The compound that registered the lowest content was the acylated form of pelargonidin-3-*O*-glucoside. Li et al. [13] suggested also that cyanidin-3-*O*-glucoside concentration was the highest in whole corn (1135.4 ± 12.9 mg/kg corn). Among the acylated ANCs, these authors suggested that the concentration of cyanidin-3-*O*-(6″-malonylglucoside) was higher than that of pelargonidin-3-*O*-(6″-malonylglucoside) and peonidin-3-*O*-(6″-malonylglucoside).

Moreover, cyanidin-based ANCs were present in greater abundance than pelargonidin- and peonidin-based anthocyanins in purple maize. Yang and Zhai [15] identified three kinds of anthocyanins extracted from the seed and cob of purple corn, accounting for 75.7%, 8.3%, and 16.0%, respectively, of the total amount of all the anthocyanins, and their retention times were 13.5, 15.7, and 16.6 min, respectively. The three major anthocyanins were cyaniding-3-*O*-glucoside, pelargonidin-3-*O*-glucoside, and peonidin-3-*O*-glucoside.

### 3.2. Degradation Kinetics of Anthocyanins from Purple Maize Flour Extract

**Figure 2:**
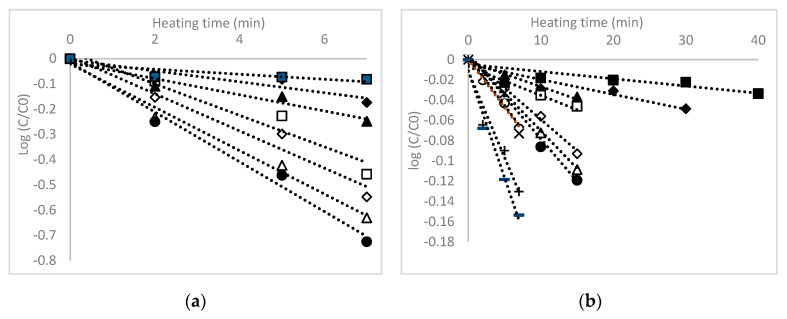
First-order kinetic model for the thermal degradation of total monomeric anthocyanin content (**a**) and antioxidant activity (**b**) in the purple maize flour extract. Temperature: ■ 80 °C, ♦ 90 °C, ▲ 100 °C, □ 110 °C, ◊ 120 °C, Δ 130 °C, ● 140 °C, ○ 150 °C, ×160 °C, +170 °C, −180 °C.

No significant heat-induced changes were found in total anthocyanins in the temperature range of 80 to 110 °C (data not shown). The degradation of ANCs between 120 and 180 °C followed a first-order model, as shown in Figure 2a. Our results are in good agreement with studies that reported the use of the first-order kinetic model for the thermal degradation of anthocyanins [22,23].

Hou et al. [22] studied the thermal stability of the four anthocyanins (cyanidin-3-*O*-glucoside, peonidin-3-*O*-glucoside, cyanidin-3,5-*O*-diglucoside, cyanidin-3-*O*-rutinoside) in black rice extract at selected temperatures (80, 90, and 100 °C) in the pH range of 1.0–6.0, suggesting a first-order reaction kinetics with respect to temperature. In the temperature range studied, the kinetic rate constants (*k*) (Table 1) for the degradation of ANCs increases with increasing temperature, with the greatest difference observed at 180 °C. Consequently, the half-life of ANCs was shorter at the higher temperature of 180 °C. Furthermore, the *k* values for the ANCs extracted from purple maize flour showed clear differences when subjected to thermal degradation at different temperatures (Table 1).

Bolea et al. [24] studied the thermal degradation of anthocyanins from different milled fractions of black rice and reported values for *k* ranging from 17.43 × 10^−2^ min^−1^ at 60 °C to 22.42 × 10^−2^ min^−1^ at 100 °C for the first fraction flour.

Sui and Zhou [23] studied the thermal stability of cyanidin-3-*O*-glucoside in an aqueous system at temperatures ranging from 100 to 165 °C, followed by the investigation of the impact of thermal treatment on the antioxidant capacity. These authors suggested a significantly lower value for the degradation rate constant of cyanidin-3-*O*-glucoside at 132.5 °C of 0.0047 s^−1^, whereas Harbourne et al. [25] reported values of 0.0028 s^−1^ for the total monomeric anthocyanins degradation at 140 °C in blackcurrant juice. The *D* values showed clear differences regarding the thermal sensitivity at different temperatures. For example, the decimal reduction time at 120 °C showed values of 101.01 ± 2.66 min, whereas an approximately 10 times decrease was observed by increasing the temperature to 180 °C (10.15 ± 0.14 min).

The *z*-value obtained confirms that the thermal resistance factors for food attributes, such as color given by related bioactives, is greater than that for spores or vegetative cells (*z* = 5–12 °C). Therefore, the rates of destruction of color are very much less temperature sensitive [26]. It is well known that the thermal degradation of ANCs starts with opening of the central ring followed by hydrolysis of the molecule, establishing colorless products. Hou et al. [22] pointed out that the stability of anthocyanins may increase via intramolecular copigmentation, especially in grain extracts, characterized by high anthocyanin content, with mixtures of different compounds, serving as copigments for intramolecular association with anthocyanins. However, these authors suggested that sugars and their degradation products tend to accelerate the degradation of anthocyanins. The Maillard reaction rate increases with the temperature, and the pigment–copigment complexes become less stable; therefore, the hydration prevails over copigmentation explaining the overall stability of the copigment on anthocyanin stability during heating [27].

The *E_a_* value calculated for the degradation of anthocyanins in the purple maize flour extract was 55.76 ± 6.83 kJ mol^−1^. Peron et al. [16] suggested values of 99.77 ± 0.87 kJ mol^−1^ in the juçara extract and 93.62 ± 0.44 kJ mol^−1^ in the “Italia” grape extract, whereas Heldman [28] previously reported that the activation energy for anthocyanin degradation ranged from 35 to 125 kJ mol^−1^.

Molecular modeling investigations were further employed to predict the events responsible for the thermal-dependent behavior of the main anthocyanins from purple maize. In agreement with the HPLC analysis, the cyanidine-3-*O*-glucoside and cyanidin-3-*O*-(6″-malonylglucoside) molecules were used for the single-molecule-level analysis. Three different starting complexes including two molecules of cyanidine-3-*O*-glucoside or cyanidin-3-*O*-(6″-malonylglucoside) were heated at 25 and 180 °C.

The simulations performed on the cyanidine-3-*O*-glucoside indicated that, when heating the complexes where the intermolecular interactions between functional groups of the flavyliums are favored (complex 1 and complex 2), the aglycons got twisted with the phenyl ring out of the plane defined by the benzopyrylium. Moreover, in the case of complex 2, the sugar moiety bended toward the aglycon, forming an angle between the two planes of the sugar moiety and of the phenyl from the flavylium structure of about 90 °C. A similar observation was reported by Dumitraşcu et al. [19] when simulating the behavior of the main anthocyanins from Cornelian cherries at temperatures of 100 and 150 °C. A different thermal behavior of the cyanidine-3-*O*-glucoside molecules was noticed when the intermolecular contacts between the sugar and the aglycon were favored in the starting complex. A close look at complex 3 revealed that at 25 °C a hydrogen bond (Hb) of 2.73 Å connected the hydroxyl groups of benzopyrylium and of C6 from the glucose moiety on the same anthocyanin molecule. Unlike complexes 1 and 2, in which heating at 180 °C resulted in the increase of the distance between the two cyanidine-3-glucoside molecules, in the case of complex 3 a tendency of aligning the benzopyrylium moieties of the two anthocyanin molecules of the complex was noticed at an intermolecular distance of ~3.9 Å. In line with these observations, the interaction energy (E) values increased with the temperature increase, suggesting lower affinity between cyanidine-3-glucoside molecules from complex 1 (*E* increased from −15.74 kcal/mol at 25 °C to −0.01 kcal/mol at 180 °C) and complex 2 (*E* increased from −16.89 kcal/mol at 25 °C to −0.02 kcal/mol at 180 °C). On the other hand, no significant variation of *E* was noticed when heating the complex 3 (*E* of −12.90 and −12.49 kcal/mol at 25 and 180 °C, respectively), suggesting that the intermolecular affinity due to the hydroxyls of benzopyrylium and of the sugar moiety is preserved even at high temperature.

Detailed analysis of the complexes made up of cyanidin-3-*O*-(6″-malonylglucoside) indicated the increase of the interaction energy with the temperature in all studied cases. The two cyanidin-3-*O*-(6″-malonylglucoside) molecules of the complex 1 equilibrated at 25 °C, established one Hb of 2.82 Å between the malonyl group on one anthocyanin and the phenyl of the second anthocyanin molecule. The attractive forces acting between the molecules are strong enough to keep them close together, even when the kinetic energy increases because of the thermal agitation of the atoms. In fact, the Hb identified at 25 °C is preserved at 180 °C (length of Hb of 2.46 Å). In a similar manner, a Hb of 3.15 Å was observed to connect the malonyl group on one anthocyanin by the hydroxyl group from C7 of the benzopyrylium in complex 2, but the temperature increase resulted in spacing out the two molecules. An interesting intramolecular copigmentation event was observed at 180 °C, consisting in glucose molecules twisting in respect to the initial model. If at 25 °C the malonyl tends to be aligned with the benzopyrylium, at 180 °C, it approaches the aromatic ring, which is carbon–carbon bound to the benzopyrylium (O9–O71 interatomic distance of 3.49 Å). The results of the molecular modeling investigations are in agreement with Nayak et al. [29] and Dumitraşcu et al. [19], indicating that the intramolecular copigmentation and intermolecular self-association might concur with anthocyanin degradation at high temperature and reduction of their color.

### 3.3. Degradation Kinetics of Antioxidant Activity from Purple Maize Flour Extract

The DPPH radical scavenging method was used in this study to evaluate the antioxidant activity of the heat-treated extract. In the un-treated state, the purple maize flour extract showed an antioxidant activity of 85.72 ± 0.73 mmol/g DW. Peron et al. [16] obtained a half maximal effective concentration (EC_50_) value for antioxidant activity of 53.9 μg/mL in juçara extract. After thermal degradation of the anthocyanin extracts at 120 °C for 30 min, there was a reduction in the expected antioxidant activity by 20%. When heating at higher temperature of 180 °C for 7 min, the reduction in antioxidant activity was of approximatively 30%. Therefore, even with this reduction and considering the intense applied time–temperature combination, it can be appreciated that the antioxidant potential of the extracts was maintained in the whole temperature range studied. The loss in antioxidants from cooked corn can be attributed to synergistic combinations or interactions of several types of chemical reactions, diffusion of water soluble compounds, and the formation or breakdown of them, as explained by Harakotr et al. [30].

Sui and Zhou [23] suggested that the thermal treatments had little impact on the overall antioxidant capacity of the anthocyanin solutions. These authors reported values for DPPH radical scavenging activity for untreated anthocyanins solutions of 0.202 mg Trolox/mL and a similar value of 0.212 mg Trolox/mL after heating at 160 °C for 30 min. The authors concluded that it is possible that the loss of antioxidant capacity through the degradation of anthocyanins is compensated by the phenolic yield.

The thermal degradation of the antioxidant activity of the purple maize flour extract also followed a first-order kinetic model (Figure 2b). Therefore, the *k* values varied from 0.16 ± 0.01 × 10^−3^ min^−1^ at 80 °C and significantly increased to 4.85 ± 0.29 × 10^−2^ min^−1^ at 180 °C (Table 2). Mercali et al. [31] also suggested that anthocyanin thermal degradation fitted a first-order reaction model with the rate constants ranging from 5.9 to 19.7 × 10^−3^ min^−1^.

The *t*_1/2_ values (Table 2) for antioxidant thermal degradation varied from 429.96 ± 13.45 min (about 7.2 h) at 80 °C to 14.26 ± 1.82 min (about 0.23 h) at 180 °C. Significant thermal differences may be observed in Table 2 when analyzing *D* values, demonstrating clear differences in thermal sensitivity at different temperatures. By increasing the temperature by 10 °C from 80 to 90 °C, the *D* value was 2 times lower, while increasing to 120 °C caused a reduction of almost 10 times. Wang and Zu [9] studied the degradation kinetics of anthocyanins in blackberry juice and concentrate and reported *t*_1/2_ values varying from 16.7 to 2.9 h for 8.9° Brix samples at 60, 70, 80, and 90 °C, whereas Cemeroğlu et al. [32] reported that *t*_1/2_ values for anthocyanins degradation were 54.3, 22.5, and 8.1 h in sour cherry juice at 60, 70, and 80 °C, respectively.

To determine the effect of temperature on the parameters studied, the *k* values were fitted to an Arrhenius-type equation. The *E_a_* value estimated for the degradation of antioxidant activity in the purple maize flour extract was 41.12 ± 3.00 kJ mol^−1^, suggesting that a higher energy is needed to thermally degrade the antioxidant activity, an aspect that can be explained by the different thermostability of the compounds from the extract responsible for the antioxidant activity.

Bolea et al. [24] suggested higher values for antioxidant activity after thermal degradation of phytochemicals from black rice flour extracts, with values ranging from 1.33 × 10^−2^ min^−1^ at 60 °C to 2.18 × 10^−2^ min^−1^ at 100 °C. Wang and Zu [9] suggested *E_a_* value of 58.95 kJ/mol for anthocyanin degradation during heating the 8.9° Brix blackberry juice, whereas Mercali et al. [31] suggested value of 74.8 kJ/mol for degradation kinetics of monomeric anthocyanins in acerola pulp during thermal treatment by ohmic and conventional heating. As expected, the *z*-value denotes that the thermal resistance of the compounds responsible for the antioxidant activity (75.75 ± 2.87 °C) is greater than that for spores or vegetative cells, suggesting that the rates of thermal destruction of bioactives are very much less temperature sensitive.

### 3.4. In Vitro Digestibility of ANCs in the Purple Maize Flour Extract as Affected by Thermal Treatment

**Figure 3:**
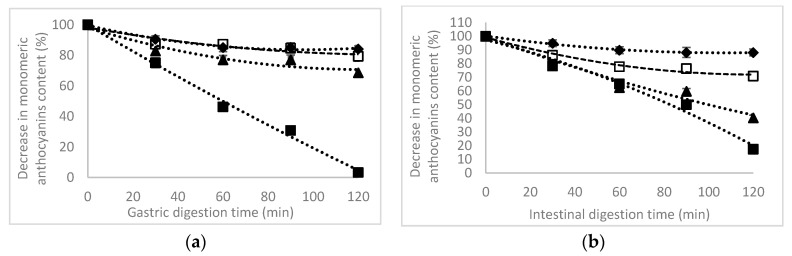
The decrease in total monomeric anthocyanins in the purple maize flour extract in gastric (**a**) and intestinal (**b**) simulated in vitro digestion as affected by thermal treatment. Temperature: ♦ 25 °C, □ 80 °C, ▲ 120 °C, ■ 180 °C.

The unheated ANCs extracts (equivalent to 10 mg C3G/mL) were digested sequentially in the gastric and intestinal simulated juices. Prior to digestion step, the extracts were heat treated at 80 °C for 40 min, and at 120 °C and 180 °C for 7 min. The TAC and antioxidant activity in the different fractions were quantified at every 30 min. During in simulated gastric digestion, the untreated TAC content showed no changes in the first 60 min of reaction, with a significant decrease of approximatively 21% after 120 min (Figure 3a).

The extract heated at 80 °C showed a high stability of ANCs at gastric digestion, with changes in ANCs during 120 min of digestion of approximatively 16%. A sequential digestion pattern was observed in the case of extract heated at 120 °C (Figure 3a), with a maximum decrease registered after 120 min of gastric digestion of 32%. When heating the extract at the higher temperature of 180 °C, the digestion ended quickly after 60 min, and low amounts of ANCs were detected after 120 min (Figure 3a). McGhie and Walton [33] suggested that the acidic conditions of the simulated gastric fluid contributed to the stability of ANCs, whereas Talavera et al. [34] reported that anthocyanin was absorbed via the stomach in rats. Thus, it can be appreciated that the high stability of the anthocyanins after gastric digestion may be very important because it suggests that in vivo, circulating metabolites may be present such as the anthocyanin metabolites found in the gastric fluid, as suggested by Kim et al. [35].

After simulated intestinal digestion, the TAC of the purple maize extract at 25 °C was slowly decreased from 5% after 30 min of digestion to a maximum of 12% after 120 min, when compared to that observed after simulated gastric digestion (Figure 3b). Heat treatment increased the degradation rate of ANCs in intestinal juice, with a maximum levels registered after 120 min of digestion of approximatively 60% after a heat treatment at 80 °C, 30% after heating at 120 °C, and 83% after a preliminary heating at 180 °C, respectively.

Regarding the mechanism of ANC metabolism, Stevens and Maier [36] reported that the process involved the opening of the intramolecular heterocyclic flavylium ring under alkaline conditions in the intestinal fraction. It is well known that ANCs are typically stable at an acidic pH but unstable at an alkaline pH. Moreover, the pH stability of anthocyanins depends on their chemical structures [30]. The methoxyl groups on the B-ring of anthocyanins seem to enhance the stability of anthocyanins at an alkaline pH. For example, it has been reported that malvidin-3-*O*-glucoside, which has methoxyl groups on the B-ring, exhibited higher stability than cyanidin-3-*O*-glucoside across the alkaline pH range [37].

### 3.5. In Vitro Digestibility of Antioxidant Activity as Affected by Thermal Treatment

**Figure 4:**
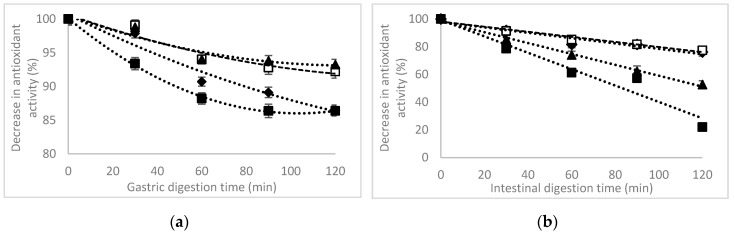
The antioxidant activity of the purple maize flour extract in gastric (**a**) and intestinal (**b**) simulated in vitro digestion as affected by thermal treatment. Temperature: ♦ 25 °C, □ 80 °C, ▲ 120 °C, ■ 180 °C.

The DPPH radical scavenging activity results of untreated extracts in simulated gastric and intestinal in vitro digestion model are presented in Figure 4.

The DPPH radical scavenging activity decreased for the untreated extract from the gastric to the intestinal fraction from approximatively 14% to 24%, respectively, after 120 min of digestion for each phase. In the gastric simulated digestion fraction, the heat treatment had no significant (*p* > 0.05) effect on DPPH radical scavenging activity, thus registering decreasing variations of approximatively 11% after heating at 80 °C, 7% after a heat treatment at 120 °C, and of 14% by heating at 180 °C, after 120 min of gastric digestion (Figure 4a). Significant decreases (*p* < 0.05) were found in intestinal simulated juice, up to 78% for the extract heated at 180 °C, whereas for the extracts with no heating or heated at 80 °C, a slow decrease up to 24% was found (Figure 4b). These changes in antioxidant activity are highly correlated with the loss of anthocyanins in gastric and intestinal fraction.

## 4. Conclusions

Purple maize extract has high anthocyanin content and high antioxidant activity, which may increase its popularity, as a significant source of bioactives. The chromatographic profile of the purple maize flour extract displayed the presence of six main compounds, namely, cyanidin-3-*O*-glucoside, pelargonidin-3-*O*-glucoside, peonidin-3-*O*-glucoside, cyanidin-3-*O*-(6″-malonylglucoside), pelargonidin-3-*O*-(6″-malonylglucoside), and peonidin-3-*O*-(6″-malonylglucoside), with the two major compounds being cyanidin-3-*O*-glucoside and its acylated form cyanidin-3-*O*-(6″-malonylglucoside). The thermostability of anthocyanins in extracts at temperatures between 80 and 120 °C was suggested, thus highlighting the opportunity to use these extracts in different processes involving high temperature–short time parameters. At temperatures ranging from 120 °C to 180 °C, both anthocyanins and antioxidant activity degraded following a first-order kinetic model. The *z*-values confirmed that the thermal resistance factors for food attributes are greater than those for spores or vegetative cells. Molecular modeling tests, performed on starting bimolecular complexes in which all types of self-association between main anthocyanins from purple corn are favored, indicated that, depending on the starting models, both intramolecular copigmentation and intermolecular self-association events are possible at 180 °C, therefore explaining the experimental results on the degradation of anthocyanins at high temperature. The thermal treatment affected the anthocyanin and antioxidant stability during the in vitro digestion. Different patterns in anthocyanins and antioxidant activity were found, both in gastric and intestinal digestion, highlighting a good stability when preliminarily heated up to 120 °C.

## Figures and Tables

**Table 1:** Kinetic parameters for the thermal degradation of total monomeric anthocyanins in the purple maize flour extract.

Temperature (°C)	*k* × 10^−2^ (min^−1^)	*D* Value (min)	*t*_1/2_ (min)
120	2.28 ± 0.024 *	101.01 ± 2.66	30.40 ± 1.24
130	4.99 ± 0.035	46.08 ± 3.72	13.86 ± 1.98
140	7.39 ± 0.125	31.15 ± 0.61	9.37 ± 0.97
150	14.37 ± 0.17	16.02 ± 0.39	4.82 ± 0.67
160	16.95 ± 0.23	13.58 ± 0.19	4.08 ± 0.45
170	19.29 ± 0.12	11.93 ± 0.25	3.59 ± 0.22
180	22.68 ± 0.20	10.15 ± 0.14	3.05 ± 0.12
*E_a_* 55.75 ± 6.83 kJ/mol (0.93)		*z*_T_ 61.72 ± 2.28 °C (0.90)	

* Standard deviation. *k*—kinetic rate constant; *D*—decimal reduction time, z_T_—the temperature-increase necessary to induce a 10-fold reduction in *D*, decimal reduction time; *t*_1/2_—time required for 50% reduction in the original content at the same temperature, *E_a_*, activation energy.

**Table 2:** Kinetic parameters for the thermal degradation of antioxidant activity of the purple maize flour extract.

Temperature (°C)	*k* × 10^−2^ (min^−1^)	*D* Value (min)	*t*_1/2_ (min)
80	0.16 ± 0.01 *	1428.57 ± 25.67	429.96 ± 13.45
90	0.34 ± 0.02	666.66 ± 17.59	200.65 ± 11.26
100	0.57 ± 0.10	400 ± 19.35	120.39 ± 16.78
110	0.64 ± 0.09	357.14 ± 8.89	107.49 ± 11.27
120	1.42 ± 0.28	161.29 ± 7.66	48.54 ± 2.24
130	1.70 ± 0.26	135.13 ± 3.82	40.67 ± 2.87
140	1.93 ± 0.25	119.04 ± 4.61	35.83 ± 2.98
150	2.18 ± 0.27	105.26 ± 2.39	31.68 ± 1.97
160	2.23 ± 0.13	103.09 ± 2.19	31.02 ± 2.67
170	3.91 ± 0.18	58.82 ± 1.12	17.70 ± 1.43
180	4.85 ± 0.29	47.39 ± 1.14	14.26 ± 1.82
*E_a_* 41.12 ± 3.00 kJ/mol (0.95)		*z_T_* 75.75 ± 2.87 °C (0.93)	

* Standard deviation.

## References

[1] Spence C. (2015). On the psychological impact of food colour. Flavour.

[2] Stevens L.J., Kuczek T., Burgess J.R., Stochelski M.A., Arnold L.E., Galland L. (2013). Mechanisms of behavioral, atopic, and other reactions to artificial food colors in children. Nutr. Rev..

[3] Somavat P., Kumar D., Singh V. (2018). Techno-economic feasibility analysis of blue and purple corn processing for anthocyanin extraction and ethanol production using modified dry grind process. Ind. Crops Prod..

[4] Assous M.T.M., Abdel-Hady M.M., Medany G.M. (2014). Evaluation of red pigment extracted from purple carrots and its utilization as antioxidant and natural food colorants. Ann. Agric. Sci..

[5] Fernandes I., Faria A., Calhau C., de Freitas V., Mateus N. (2014). Bioavailability of anthocyanins and derivatives. J. Funct. Food.

[6] Esposito D., Damsud T., Wilson M., Grace M.H., Strauch R., Li X., Lila M.A., Komarnytsky S. (2015). Black currant anthocyanins attenuate weight gain and improve glucose metabolism in diet-induced obese mice with intact, but not disrupted, gut microbiome. J. Agric. Food Chem..

[7] He F., Liang N., Mu L., Pan Q., Wang J., Reeves M.J., Duan C. (2012). Anthocyanins and their variation in red wines I. Monomeric Anthocyanins and their color expression. Molecules.

[8] Flores G., del Castillo M.L.R., Costabile A., Klee A., Bigetti Guergoletto K., Gibson G.R. (2015). *In vitro* fermentation of anthocyanins encapsulated with cyclodextrins: Release, metabolism and influence on gut microbiota growth. J. Funct. Food.

[9] Wang W.-D., Xu S.-Y. (2007). Degradation kinetics of anthocyanins in blackberry juice and concentrate. J. Food Eng..

[10] Patras A., Brunton N.P., O’Donnell C., Tiwari B.K. (2010). Effect of thermal processing on anthocyanin stability in foods; mechanisms and kinetics of degradation. Trends Food Sci. Technol..

[11] Costa H.C.B., Silva D.O., Vieira L.G.M. (2018). Physical properties of açai-berry pulp and kinetics study of its anthocyanin thermal degradation. J. Food Eng..

[12] Abdel-Aal E.-S.M., Young J.C., Rabalski I. (2006). Anthocyanin composition in black, blue, pink, purple, and red cereal grains. J. Agric. Food Chem..

[13] Li Q., Somavat P., Singh V., Chatham L., Gonzalez de Mejia E. (2017). A comparative study of anthocyanin distribution in purple and blue corn coproducts from three conventional fractionation processes. Food Chem..

[14] Giusti M.M., Worsltad R.E. (2001). Characterization and measurement of anthocyanins by UV–visible spectroscopy. Current Protocols in Food Analytical Chemistry New York.

[15] Yang Z., Zhai W. (2010). Identification and antioxidant activity of anthocyanins extracted from the seed and cob of purple corn (*Zea mays* L.). Innov. Food Sci. Emerg. Technol..

[16] Peron D.V., Fraga S., Antelo F. (2017). Thermal degradation kinetics of anthocyanins extracted from juçara (*Euterpe edulis* Martius) and ‘‘Italia” grapes (*Vitis vinifera* L.), and the effect of heating on the antioxidant capacity. Food Chem..

[17] Lee S.-J., Lee S.Y., Hur S.J. (2015). Effect of Escherichia coli and Lactobacillus casei on luteolin found in simulated human digestion system. J. Food Nutr. Res..

[18] Castaneda-Ovando A., de Lourdes Pacheco-Hernández M., Páez-Hernández M.E., Rodríguez J.A., Galán-Vidal C.A. (2009). Chemical studies of anthocyanins: A review. Food Chem..

[19] Dumitraşcu L., Enachi E., Stănciuc N., Aprodu I. (2019). Optimization of ultrasound assisted extraction of phenolic compounds from cornelian cherry fruits using response surface methodology. CyTA-J. Food.

[20] Pascual-Teresa S., Santos-Buelga C., Rivas-Gonzalo J.C. (2002). LC-MS analysis of anthocyanin from purple corn cob. J. Sci. Food Agric..

[21] Saikaew K., Lertrat K., Meenune M., Tangwongchai R. (2018). Effect of high-pressure processing on colour, phytochemical contents and antioxidant activities of purple waxy corn (*Zea mays* L. var. *ceratina*) kernels. Food Chem..

[22] Hou Z., Qin P., Zhang Y., Cui S., Ren G. (2013). Identification of anthocyanins isolated from black rice (*Oryza sativa* L.) and their degradation kinetics. Food Res. Int..

[23] Sui X., Zhou W. (2014). Monte Carlo modelling of non-isothermal degradation of two cyanidin-based anthocyanins in aqueous system at high temperatures and its impact on antioxidant capacities. Food Chem..

[24] Bolea C.A., Grigore-Gurgu L., Aprodu I., Vizireanu C., Stănciuc N. (2019). Process-Structure-Function in Association with the Main Bioactive of Black Rice Flour Sieving Fractions. Foods.

[25] Harbourne N., Jacquier J.C., Morgan D.J., Lyng J.G. (2008). Determination of the degradation kinetics of anthocyanins in a model juice system using isothermal and non-isothermal methods. Food Chem..

[26] Holdsworth S.D. (1985). Optimisation of thermal processing A review. J. Food Eng..

[27] Gradinaru G., Biliaderis C.G., Kallithraka S., Kefalas P., Garcia-Viguera C. (2003). Thermal stability of *Hibiscus sabdariffa* L. anthocyanins in solution and in solid state: Effects of copigmentation and glass transition. Food Chem..

[28] Heldman D.R. (2011). Food preservation process design. Advances in Food Process Engineering Research and Applications.

[29] Nayak B., Berrios J.D.J., Powers J.R., Tang J. (2011). Thermal degradation of anthocyanins from purple potato (cv. Purple Majesty) and impact on antioxidant capacity. J. Agric. Food Chem..

[30] Harakotr B., Suriharn B., Tangwongchai R., Scott M.P., Lertrat K. (2014). Anthocyanin, phenolics and antioxidant activity changes in purple waxycorn as affected by traditional cooking. Food Chem..

[31] Mercali G.D., Jaeschke D.P., Tessaro I.C., Ferreira Marczak L.D. (2013). Degradation kinetics of anthocyanins in acerola pulp: Comparison between ohmic and conventional heat treatment. Food Chem..

[32] Cemeroğlu B., Velioğlu S., Isik S. (1994). Degradation kinetics of anthocyanins in sour cherry juice and concentrate. J. Food Sci..

[33] McGhie T.K., Walton M.C. (2007). The bioavailability and absorption of anthocyanins: Towards a better understanding. Mol. Nutr. Food Res..

[34] Talavera S., Felgines C., Texier O., Besson C., Lamaison J.-L., Rémésy C. (2003). Anthocyanins are efficiently absorbed from the stomach in anesthetized rats. J. Nutr..

[35] Kim I., Moon J.K., Hur S.J., Lee J. (2020). Structural changes in mulberry (*Morus Microphylla*. Buckl) and chokeberry (*Aronia melanocarpa*) anthocyanins during simulated in vitro human digestion. Food Chem..

[36] Stevens J.F., Maier C.S. (2016). The chemistry of gut microbial metabolism of polyphenols. Phytochem. Rev..

[37] Loypimai P., Moongngarm A., Chottanom P. (2016). Thermal and pH degradation kinetics of anthocyanins in natural food colorant prepared from black rice bran. J. Food Sci. Technol..

